# Equivalent square formula for determining the surface dose of rectangular field from 6 MV therapeutic photon beam

**DOI:** 10.1120/jacmp.v14i5.4340

**Published:** 2013-09-06

**Authors:** Lukkana Apipunyasopon, Somyot Srisatit, Nakorn Phaisangittisakul

**Affiliations:** ^1^ Department of Nuclear Engineering Faculty of Engineering Chulalongkorn University Bangkok Thailand; ^2^ Department of Physics Faculty of Science Chulalongkorn University Bangkok Thailand; ^3^ ThEP Center, CHE 328 Si‐Ayuttaya Road Bangkok Thailand

**Keywords:** surface dose, build‐up dose, rectangular field, equivalent square, Monte Carlo simulation

## Abstract

The purpose of the study was to investigate the use of the equivalent square formula for determining the surface dose from a rectangular photon beam. A 6 MV therapeutic photon beam delivered from a Varian Clinac 23EX medical linear accelerator was modeled using the EGS4nrc Monte Carlo simulation package. It was then used to calculate the dose in the build‐up region from both square and rectangular fields. The field patterns were defined by various settings of the X‐ and Y‐collimator jaw ranging from 5 to 20 cm. Dose measurements were performed using a thermoluminescence dosimeter and a Markus parallel‐plate ionization chamber on the four square fields (5 × 5, 10 × 10, 15 × 15, and $20\times 20\;{\text{cm}}^{2}$). The surface dose was acquired by extrapolating the build‐up doses to the surface. An equivalent square for a rectangular field was determined using the area‐to‐perimeter formula, and the surface dose of the equivalent square was estimated using the square‐field data. The surface dose of square field increased linearly from approximately 10% to 28% as the side of the square field increased from 5 to 20 cm. The influence of collimator exchange on the surface dose was found to be not significant. The difference in the percentage surface dose of the rectangular field compared to that of the relevant equivalent square was insignificant and can be clinically neglected. The use of the area‐to‐perimeter formula for an equivalent square field can provide a clinically acceptable surface dose estimation for a rectangular field from a 6 MV therapy photon beam.

PACS number: 87.55.ne

## I. INTRODUCTION

High‐energy photons generated by a medical linear accelerator have been commonly used in radiotherapy. With an increasing energy of ionizing radiation, the penetrating power of photons and secondary electrons increases, leading to a deeper position of the point of maximum dose. However, the accumulated dose at the boundary between the air and patient's skin, which is known as the surface dose, is not clinically negligible and should be taken into account with the treatment plan in order to spare the normal skin. The surface dose can become a limiting factor when deciding on the required dose for a deep‐seated tumor. As a result, knowing the accurate surface dose is imperative for assessing the skin damage, as well as designing an appropriate irradiation technique and scheme of dose fractionation.

For high‐energy photon beams, the dose deposited within the first few millimeters of skin depth varies considerably due to the contaminant electrons.^(^ ^1^ ^,^ ^2^ ^)^ They are mostly originated from the interaction of photons with components of the accelerator head, especially, the flattening filter.^(1)^ Relatively small portion of the contaminant electron occurred in an air column and in a phantom.^(2)^ To measure the dose in the build‐up region, the size of the dosimeter along the beam direction should be as small as possible due to the high dose gradient. A typical cylindrical chamber, as commonly used in the depth dose measurement, is not suitable for the accurate measurement of build‐up dose. Several more appropriate dosimeters have been used to measure the dose in the build‐up region of therapeutic photon beams, such as a thermoluminescense dosimeter (TLD),^(3)^ radiochromic film,^4^, ^5^, ^6^ extrapolation chamber,^7^, ^8^, ^9^ parallel‐plate ionization chamber.^(^ ^4^ ^,^ ^6^ ^,^ ^9^ ^,^ ^10^ ^)^ Among these, the extrapolation chamber gives the most accurate results and is recommended for the reliable measurement of the dose in the build‐up region.^(^ ^8^ ^,^ ^9^ ^)^ Using the extrapolation chamber, the ionization per unit volume at the surface is measured as a function of the electrode spacing, and the surface dose is estimated from the data extrapolation to zero electrode spacing. Unfortunately, few institutions have access to this instrument and, in addition, the procedure for such measurements with it is time‐consuming. Therefore, the accurate measurement of the surface dose is impractical in a clinical setting.

The Monte Carlo (MC) simulation has been demonstrated to be an accurate method for dose calculation in radiotherapy since the beam's particles are tracked individually in the media according to a reliable interaction database. MC calculations have been benchmarked and validated by various investigators^(11)^ and often serve as the gold standard in many situations. Moreover, an excellent agreement on the build‐up dose between those derived from MC simulations and the measurements using an extrapolation chamber have been found previously.^(9)^

Typically, several dose parameters from a medical linear accelerator are collected for the square field such as the percentage depth dose, tissue‐air ratio, head scatter factor, and output factor. For the purposes of dose calculation for a rectangular field, it is customary to find its equivalent square for a specific dose parameter. For example, the head scatter factor from a given rectangular field can be related to the square field by the well‐established equivalent square relationship, in the form of the equivalent‐square table,^(12)^ the area‐to‐perimeter (A/P) formula,^(13)^ and the geometric formula.^(14)^ The tables of equivalent squares, calculated from the integration of the scatter‐radius function, have been introduced by Day and Aird.^(12)^ In the A/P formalism,^(13)^ the side of an equivalent square is calculated by:

(1) L=4A/P

where *A* and *P* are the area and the perimeter of the rectangular field, respectively. Evidently, this formalism gives the two different rectangular fields with the same area and perimeter, $L_{x}\;\times \;L_{y}$ and $L_{y}\;\times \;L_{x}$, the same side of an equivalent square. Such two fields can be obtained by simply interchanging the setting of X and Y collimator; therefore, this is known as the collimator exchange. Kim et al.^(14)^ has proposed the geometric formula which accounts for both the effect of field elongation and collimator exchange based on the linac head's geometry. Practically, the equivalent square formula is preferred due to its simplicity, as long as it accurately predicts the required dose parameter. Additionally, since the contaminant electrons majorly come from the flattening filter,^(1)^ as a result, the collimator exchange effect on the surface dose should be less critical than in that of the head scatter factor. Therefore, the A/P formalism is conceptually expected to better correlate the surface dose of the rectangular field to that of the square field than in the case of the head scatter factor. However, the validation study of the equivalent square approach for predicting the surface dose from a rectangular photon beam is very limited. To our knowledge, the study by Gosselin et al.,^(10)^ in which Day's tables were used and the measurements were done using plane‐parallel ionization chamber, is the only such study.

In this study, the surface doses of a 6 MV photon beam from Varian Clinac 23EX with various square and rectangular fields were estimated with the EGS4nrc MC simulation. In order to correlate the surface dose of rectangular field to that of the square field, the side of the equivalent square of the rectangular field was computed using the A/P relationship. The surface dose of the equivalent square field was then estimated from a linear interpolation of the square‐field surface doses. Subsequently, the surface dose of the rectangular field was compared with that of the relevant equivalent square field to see whether the equivalent square field approach is suitable for the surface dose's approximation of the rectangular field from the 6 MV photon beam.

## II. MATERIALS AND METHODS

The EGS4nrc MC simulation package^(15)^ was employed to model a 6 MV photon beam from the Varian Clinac 23EX medical linear accelerator (Varian Medical Systems, Palo Alto, CA) and to calculate the dose distribution in a water phantom. Specifically, the BEAMnrc code^(^ ^16^ ^,^ ^17^ ^)^ was used to model the linac's head components, which consists of the target, primary collimator, vacuum window, monitoring chamber, mirror, secondary collimator, and multileaf collimator. The description of the dimensions, geometries, and materials of all components were taken from the manufacturer's detailed specifications. To calculate the dose distribution in the phantom, the DOSXYZnrc code^(^ ^16^ ^,^ ^17^ ^)^ was used.

The transport parameters used in the BEAMnrc for generating the phase‐space files and in the DOSXYZnrc for dose calculation were as follows: ECUT = 700 keV,PCUT = 10 keV,AE = 700 keV, and AP = 10 keV The particle's transport is terminated and its residual energy is transferred in the current region when the total energies of the electron and photon are less than the value of ECUT and PCUT, respectively. The production of secondary particles is considered if the particle's total energy is greater than AE for the knock‐on electrons and greater than AP for the bremsstrahlung photons. To specifically calculate the surface dose, both the ECUT and AE values were lowered to 521 keV The values of the transport parameters were selected to ensure the accuracy of the computed dose to be better than 1%.^(^ ^18^ ^,^ ^19^ ^)^ The phase‐space file, which contains the information on each beam's particle, was recorded on the plane perpendicular to the beam axis at 90 cm from the source.

Using the phase space file obtained from the BEAMnrc code as the input for the DOSXYZnrc code, the beam's interaction in the $30\;\times \;30\;\times \;20\;{\text{cm}}^{3}$ water phantom was simulated and from this the deposited dose within a defined voxel was obtained. For the depth dose, the voxel size was made thinner around the depth of maximum dose, $1\;\times \;1\;\times \;0.2\;{\text{cm}}^{3}$ and it was thicker at other depth (i.e., 0.5–1.0 cm). For the lateral dose profile, the voxel size was made smaller in the penumbra region $(0.4\;\times \;1\;\times \;1\;{\text{cm}}^{3}$) than in the in‐beam region $(1\;\times \;1\;\times \;1\;{\text{cm}}^{3}$).

Since the actual information about the incident electron beam on the target inside the linac was experimentally unknown in this study, it was modeled as a monoenergetic circular beam with a Gaussian distribution along its radial dimension. The energy and the full‐width‐at‐half‐max (FWHM) of the radial distribution of the electron beam were determined from the best match between the simulated and measured results for the percentage depth dose along the central axis and the beam profiles at a 10 cm depth for photon beam field sizes of 10 × 10 and $30\times 30\;{\text{cm}}^{2}$. The energy of the incident primary electrons was varied from 6.0 to 6.5 MeV, while the FWHM of the radius distribution was investigated over the range from 1.0 to 1.6 mm.

A cylindrical ionization chamber of type CC13 (Markus 23392, PTW‐Freiburg, Germany), with an active diameter of 6 mm and a cavity volume of $0.13\;{\text{cm}}^{3}$, was used to measure the depth dose in the water phantom. Since the measured dose in the build‐up region using this detector is uncertain, the matching condition on the percentage depth dose started from the depth at a maximum dose (about 1.5 cm) down to 30 cm. For the lateral beam profiles, a silicon p‐type photon semiconductor dosimeter of type PFD (Scanditronix Wellhofer, Germany) was utilized. This has an active diameter of 2 mm and an effective thickness of 0.06 mm from the detector's front surface. The matching ranges for the beam profile were from ‐14 to +14 cm and from ‐29 to +29 cm for a field size of 10 × 10 and $30\times 30\;{\text{cm}}^{2}$, respectively. The agreement between the simulated and measured data was examined according to the maximum difference between the two datasets at the same position within the matching range and also the average difference.

The field size in this study was defined by the secondary X and Y collimator. The sizes of the square field were 5 × 5, 10 × 10, 15 × 15, and $20\times 20\;{\text{cm}}^{2}$. The rectangular fields were obtained by: (i) the X collimator was fixed such that it produced a 20 cm opening, while the Y collimator was varied in the way that it gave the field opening of 5, 8, 10, 12, 15, 18, and 20 cm; and (ii) the previous field patterns were repeated in which the Y collimator was fixed and the X collimator was varied. In other words, the two sets of rectangular field were obtained from the exchange between the X and Y collimators.

For the MC determination of the build‐up dose, the voxel size along the beam central axis from the phantom's surface to a few millimeters depth was set to $3\;\times \;3\;\times \;0.014\;{\text{cm}}^{3}$, while it was $1\;\times \;1\;\times \;0.014\;{\text{cm}}^{3}$ for the fields with 5 cm side. The number of simulated primary electrons was in the order of $10^{9}$, so that a standard statistical uncertainty of less than 1% of the deposited dose in the above voxel was achieved.

For each field size, the absorbed dose at the surface normalized to the maximum dose, called the surface dose, was obtained by extrapolating the percentage doses at a depth of less than 0.3 cm to the zero depth using a third‐order polynomial function. Based on the surface doses of the four square fields, the correlation between the side of the square field and the surface dose was then examined by the method of least‐square fitting.

Since the extrapolation chamber was not available to us, the measurements for the central axis depth dose were done using a parallel‐plate ionization chamber (Markus 23392, PTW‐Freiburg, Germany) and a TLD (HARSHAW Chemical Co, Solon, OH). These two dosimeters have often been used in the build‐up region because of their small size. The plate separation of the Markus chamber is 2 mm, with a 0.35 mm distance between the side wall and the collector. Its effective measured point was assumed to be at the bottom of the entrance window electrode. Each measured signal was taken from an average of five readings from an output variation acquired by the DOSE1 Electrometer (IBA Dosimetry, Schwarzenbruck, Germany). In order to take into account the polarity effects, the measurements with both a positive and a negative 300 V voltage were performed and the polarity correction factor was found to be 0.98. As the result, the uncertainty of our ionization chamber measurement was estimated to be less than about 2%.

The TLDs used here were a lithium fluoride (LiF) crystal doped with magnesium and titanium (thickness of 0.39 mm and surface area of $3.15\;\times \;3.15\;{\text{mm}}^{2}$). The effective point of measurement for the TLD was assumed to be at the middle of its thickness. Three repeated measurements were made to obtain the dose at each depth, which gave the TLD uncertainty to be about 3%. The variation of the TLD response with respect to the changes in either the field size or the measurement location was not included in this study, since the effect of photon spectral variations on the response is reported to be less than 1% for all of the dose measurements.^(20)^

The measurements for the square fields at 100 cm SSD were performed in a $30\;\times \;30\;\times \;20\;{\text{cm}}^{3}$ solid water equivalent phantom slab (Model‐457 (Gammex RMI, Giessen, Germany), density $1.4\;g/{\text{cm}}^{3}$) at a depth of 0, 0.2, 0.3, 0.5, 1, 1.2, 1.5, 2, and 3 cm.

For the rectangular field, the side of its equivalent square, $L_{\text{eq}}$, was calculated using the A/P relationship:^(11)^

(2) $$L_{\text{eq}}=2\;L_{x}\;L_{y}\;/(L_{x}+L_{y})$$

where $L_{x}$ and $L_{y}$ are the field's side as defined by the X and Y collimators, respectively. Obviously, $L_{\text{eq}}$ is invariant under an interchange between $L_{x}$ and $L_{y}$. Therefore, this formula does not include the effect of collimator exchange. The surface dose of the equivalent square $L_{\text{eq}}$ was then obtained using the correlation between the side of the square field and the surface dose. Subsequently, the surface dose of the rectangular field was compared with that of the equivalent square field.

## III. RESULTS

**Figure 1:**
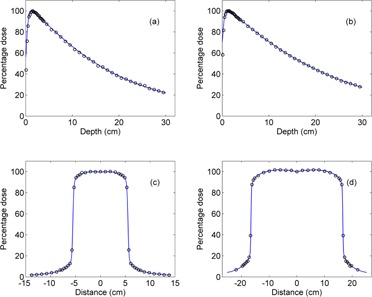
The dose distributions in a water phantom from a 6 MV photon beam: the percentage depth dose of (a) $10\times 10\;{\text{cm}}^{2}$ and (b) $30\times 30\;{\text{cm}}^{2}$ field, and the dose profile at 10 cm depth of (c) $10\times 10\;{\text{cm}}^{2}$ and (d) $30\times 30\;{\text{cm}}^{2}$ field. Solid line and open circle represent the measured and simulated data, respectively. In the MC simulation, the energy of the electron beam incident on target is 6.1 MeV with a Gaussian distribution of the radius of 1.2 mm. The standard statistical uncertainty of MC data is less than 2%, and the uncertainty bars for both measured and simulated data are smaller than the symbol size.

In the MC simulation, the primary electron beam incident on the X‐ray target was modeled with the energy of 6.1 MeV and the FWHM of the Gaussian radial distribution of 1.2 mm, since it produced the best matching of depth dose curves and dose profiles with the measurement in the water phantom for the 6 MV photon beam from our Varian Clinac 23EX machine (Fig. 1).

**Figure 2:**
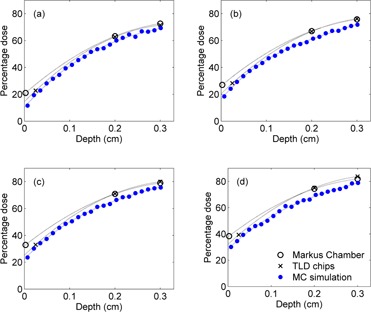
The percentage depth doses in the build‐up region for a square field size of (a) 5 × 5, (b) 10 × 10, (c) 15 × 15, and (d) $20\times 20\;{\text{cm}}^{2}$, obtained from the Markus chamber, the TLD chips, and the MC simulation. The uncertainty bars of about 2% for both measured and simulated data are smaller than the symbol.

**Figure 3:**
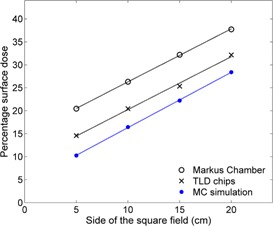
The percentage surface dose as a function of the side of the square field from both measurements (using Markus chamber and TLD chips), and the MC simulation of the four different square field sizes. The uncertainty of each data point is less than 2% and the uncertainty bars are smaller than the symbol.

The build‐up dose of each of the four square fields was calculated by the MC simulation and was also measured using the Markus chamber and the TLD (Fig. 2). The trend of the measured build‐up dose from both dosimeters was similar to that of the simulated dose. However, the measured data are always greater than the simulated data. The percentage surface dose $\left(D_{0}\right)$ was obtained by extrapolating the percentage build‐up dose to zero depth. Linear relationship between the percentage surface dose and the side of the square field (correlation coefficient $R^{2}\;\sim \;1$) was observed in the MC‐based theoretical and both empirically determined methods (Fig. 3). The percentage surface dose obtained from the TLD and the Markus chamber for all square fields was larger than that from the simulation by approximately 4% and 10%, respectively. In other words, subtracting the percentage surface dose $D_{0}$ obtained from the TLD and the Markus chamber by 4 and 10, respectively, will approximately yield $D_{0}$ derived from the MC data. From the MC data, $D_{0}$ increased linearly from about 10% to 28% as the square's side increased from 5 to 20 cm and was correlated to the side of the square field ($L_{\text{sq}}$) in cm, as shown in Eq. (3):

(3) $$D_{0}(L_{\text{sq}})[\%]=(1.204[\%\;/\text{cm}])L_{\text{sq}}[\text{cm}]+4.290\%$$

**Figure 4:**
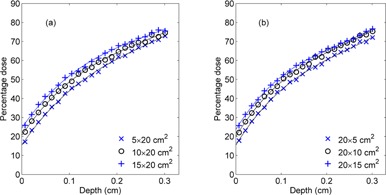
The percentage depth dose in the build‐up region for some of the different rectangular fields considered in this study. The standard statistical uncertainty of MC data is less than 2% and the uncertainty bars are smaller than the symbol.

The build‐up dose of the rectangular field increased with increasing field size, whilst the collimator exchange effect was not obvious (Fig. 4 and Table 1). The equivalent square of each rectangular field was determined by the A/P formula, using Eq. (2), and its corresponding percentage surface dose, $D_{0}$ ($L_{\text{eq}}$), was calculated using Eq. (3) and compared with its actual value (Table 1).

**Table 1:** The percentage surface dose $\left(D_{0}\right)$ of the rectangular field patterns, as determined by MC simulation and in comparison with that of its equivalent square

$L_{x}(\text{cm})$	$L^{y}\;(\text{cm})$	*Surface Dose (%)*	*Side of Equivalent Square* $L_{\text{eq}}\;(\text{cm})$ ^*a*^	*Estimated Surface Dose (%)* ^*b*^	*Difference of Surface Dose (%)*
5	20	15.56	8.00	13.92	1.61
20	5	15.53			1.64
8	20	18.67	11.43	18.05	0.62
20	8	19.49			1.44
10	20	20.63	13.33	20.34	0.29
20	10	20.08			‐0.26
12	20	22.56	15.00	22.35	0.21
20	12	22.28			‐0.07
15	20	24.47	17.14	24.93	‐0.46
20	15	23.68			‐1.25
18	20	25.63	18.95	27.10	‐1.47
20	18	25.36			‐1.74

a Using Eq. (2)

b Using Eq. (3)

## IV. DISCUSSION

For a high‐energy photon beam, as used in conventional radiotherapy, the surface dose may be of less concern because of the skin‐sparing effect, which allows high‐energy photons to be delivered to deep‐seated tumors without damaging the skin. However, for unconventional hypofractionated radiation where the fractional dose is extremely high, an acute skin reaction can occur. Therefore, the surface dose becomes one of the important factors in radiation treatment planning. To accurately measure the surface dose, the extrapolation ionization chamber, which is rarely available in most institutes, is recommended. Unfortunately, to our knowledge, no study has ever been reported on the validity of using the extrapolation chamber to obtain the surface dose of a rectangular field.

MC simulation has been shown to be an alternative, reliable method to accurately determine the surface dose.^(9)^ For the first time, here, the MC simulation has been used to investigate the surface dose of a rectangular field from a therapeutic photon beam and, also, the equivalent square formula (instead of Day's tables) has been applied.

The results from both measurements and the MC simulation were in broad agreement and showed that the surface dose increases linearly with the side of the square field, at least over the range from 5 to 20 cm, which is consistent with previous studies.^(^ ^6^ ^,^ ^9^ ^,^ ^10^ ^)^ The measured surface dose was found to be larger than that obtained by the MC simulation, which is also consistent with previous reports.^(^ ^9^ ^,^ ^10^ ^)^ This overestimation is caused by the lack of equilibrium of charged particles in the build‐up region and the finite size of the dosimeter.

For each rectangular field pattern, the interchange between the X and Y collimator (e.g., $L_{x}\;\times \;L_{y}$ vs. $L_{y}\;\times \;L_{x}$), resulted in only a minor difference (less than 1%) in the percentage surface dose (Table 1). This is because the major contribution to the surface dose from the photon beam is the contaminant electron which is mainly originated at the flattening filter.^(1)^ The effect of collimator exchange on the surface dose is, therefore, practically unimportant and can be essentially excluded in the equivalent square formula for the surface dose determination.

The percentage surface doses of the rectangular field and of its equivalent square field using the A/P relationship were in good agreement with a maximum difference of less than 2%. For the surface dose, the magnitude of this difference is considered to be small and may be clinically neglected. Contrary to the past study using the plane‐parallel ionization chamber and the tables of equivalent squares,^(10)^ the estimated surface dose obtained here using the MC simulation and the A/P formula was not always underestimated.

In this study, the side of the square field in which the surface dose is available ranged from 5 to 20 cm. As a result, the surface dose of the rectangular field, in which its equivalent square side is out of that range, cannot be determined. In addition, there are other more elongated fields in which their equivalent square fall within our range, especially those having a side of greater than 20 cm, but they are not investigated here. The effect of a multileaf collimator was not taken into account in these MC simulations since our fields were defined by the collimators. Nevertheless, significant discrepancy from the square‐field data is not expected since, again, the major contribution to the surface dose is the contaminant electron generated mainly at the flattening filter, not at the collimators.^(1)^

## V. CONCLUSIONS

To estimate the surface dose of a rectangular field from the square‐field data, the equivalent square formula is suggested. Since the collimator exchange effect does not significantly influence the surface dose, the A/P relationship is appropriate, and its applicability to determine the surface dose from rectangular photon beam is satisfactory.

## ACKNOWLEDGMENTS

The authors would like to thank sincerely the reviewers who have given very useful comments and suggestions that helped to improve the manuscript. This work is supported by The 90th Anniversary of Chulalongkorn University Fund and Special Task Force for Activating Research (STAR), Ratchadaphiseksomphot Endowment Fund. We also acknowledge the support from the National Research Council of Thailand.
